# Topical applications of allogeneic adipose-derived mesenchymal stem cells ameliorate the canine keratoconjunctivitis sicca

**DOI:** 10.1186/s12917-022-03303-7

**Published:** 2022-06-10

**Authors:** Li-Ning Wei, Ching-Ho Wu, Chung-Tien Lin, I-Hsuan Liu

**Affiliations:** 1 Institute of Veterinary Clinical Sciences, School of Veterinary Medicine, National Taiwan University, Taipei, 106 Taiwan; 2 Department of Ophthalmology, National Taiwan University Veterinary Hospital, Taipei, 106 Taiwan; 3 Department of Small Animal Surgery, National Taiwan University Veterinary Hospital, Taipei, 106 Taiwan; 4 Department of Animal Science and Technology, National Taiwan University, Taipei, 106 Taiwan; 5 Research Center for Developmental Biology and Regenerative Medicine, National Taiwan University, Taipei, 106 Taiwan

**Keywords:** Adipose-Derived Mesenchymal Stem Cells, Dry Eye Syndromes, Keratoconjunctivitis Sicca, Ophthalmic Administration

## Abstract

**Background:**

Canine keratoconjunctivitis sicca (KCS) is predominantly an immune-mediated disease. Current therapy of canine KCS is mainly by immunosuppressant, but the effectiveness was limited in some patients. In the past few years, some studies showed the results of the use of mesenchymal stem cells in treating canine KCS via periocular injections. However, the periocular injection procedure requires sedation or general anesthesia, and may lead to iatrogenic or incidental injury during the injection process. The aim of this study was to investigate the efficacy of topical allogenic canine adipose-derived mesenchymal stem cells (cAD-MSCs) in clinical patients of canine KCS.

**Results:**

The cAD-MSCs used in this study were characterized for their capability of tri-lineage differentiation and immunomodulatory properties. In addition, preparation methods for eye drops of cAD-MSCs was developed and its optimal preservation was tested. The canine KCS patients were recruited for clinical trial and divided into two groups based on their history of previous treatment. All patients received topical cAD-MSCs treatment once per week for 6 consecutive weeks and complete ophthalmic examinations were performed 1 week before treatment (week 0) and at 3rd, 6th, 9th weeks, respectively. The results showed that the quantity and quality of tears have improved significantly following topical cAD-MSCs treatment based on Schirmers tear test-1 and tear break-up time. More than half of all patients were found improved in the tear quantity. In particular, 56.5% of the patients that were unresponsive to prior immunosuppressant therapy had an effective increase in tear volume. The severity of clinical signs was also ameliorated according to the numeric rating scale score from both patient owners and the clinician.

**Conclusion:**

To sum up, topical cAD-MSCs may be beneficial especially in KCS patients with poor owner compliance for frequent daily use of eye drops or those who are unresponsive to immunosuppressant therapy.

## Background

Keratoconjunctivitis sicca (KCS), or dry eye syndrome (DES), is one of the commonly recognised ocular disease in human and canine patients. KCS is characterized by the development of ocular surface damage, and is literally an inflammatory condition of the cornea and conjunctiva, which is secondary to a deficiency of the pre-corneal tear film. The tear film not only protects and lubricates the eyes, but also provides the cornea with the oxygen and nutrients [1]. Accordingly, the tear film deficiencies may lead to dehydration and malnutrition of the corneal and conjunctival epithelium and consequently recurrent corneal ulceration and infection. As a result, the eyes show clear to yellowish discharge with corneal scarring, neovascularization, hyperpigmentation, hyperkeratinization, and may lead to vision reduction or even loss in the end stage.

In principle, KCS is categorized to quantitative KCS and qualitative KCS by tear film deficiency. Quantitative KCS is a decrease or lacking in the aqueous component of the tear film and is more common found in veterinary medicine. Although the causes of KCS in canine patients include congenital, metabolic, infectious, drug induced, neurogenic, radiation and iatrogenic, the predominantly most common cause is the idiopathic KCS (iKCS). In the recent decades, iKCS is generally considered to have an immunological and likely an autoimmune cause [2]. Therefore, more and more studies used the term “immune-mediated” instead of “idiopathic” KCS for iKCS. It is now believed that canine KCS is predominantly a T-cell immune-mediated condition as most inflammatory cells in the lacrimal tissue of KCS-affected dogs were CD3+ T cells [3]. This T-cell mediated reaction is likely compromise the tissue integrity and functionality of lacrimal glands and in turn causes KCS. Accordingly, current primary medical therapy against KCS usually includes immunosuppressants.

The first topical immunosuppressant used in dogs was cyclosporine A (CsA), and a series of studies showed that CsA blocks normal production of interleukin-2, thereby inhibits proliferation of T-helper and cytotoxic T cells in the lacrimal gland, and consequently results in marked regression of chronic corneal neovascularization and pigmentation with rapid healing of indolent corneal ulcers [4–6]. CsA is now commercially available as 0.2% ointment (Optimmune, Merck Animal Health, USA) in veterinary medicine. However, CsA can cause hypersensitivity to the eyes in some patients and is less effective especially in more advanced canine KCS. Alternatively, topical application of aqueous suspension of tacrolimus twice daily might be used as a replacement in the patients that did not respond well to CsA [7, 8]. With similar molecular mechanism to tacrolimus, twice daily eye drops of immunosuppressant pimecrolimus also showed better therapeutic effect than CsA [9]. Unfortunately, there are still a significant number of canine KCS patients do not respond to immunosuppressant treatment. Furthermore, chronic topical immunosuppressive therapy for KCS may lead to risks for other complications such as corneal and conjunctival infections and corneal squamous cell carcinoma dogs [10, 11].

Due to its multipotential differentiation capability, mesenchymal stem cells (MSCs) have attracted attention for regenerative medicine in the past two decades. According to the International Society for Cellular Therapy, the minimal criteria for cells to be recognized as MSCs include: (1) adherence to plastic in standard culture conditions; (2) specific surface antigen expression. More precisely, 95% above of MSCs must express CD73, CD90 and CD105 and 2% below of MSCs must lack expression of CD34, CD45, CD11b or CD14, CD79α or CD19 and HLA-DR, that is an MHC class II cell surface receptor; and (3) multipotent differentiation potential. Under standard conditions, the MSCs must be able to differentiate to osteoblasts, adipocytes and chondroblasts in vitro [12]. Interestingly, instead of the differentiation potentials of mesenchymal stem cells in cell replacement, current research on MSCs is focused on its immunological properties, paracrine effect, and MSC homing in cell empowerment [13].

Recent studies indicated that MSCs exert immunomodulatory effects via multiple mechanisms including indoleamine-pyrrole 2,3-dioxygenase (IDO) via adenosine signaling, prostaglandin E2 (PGE2) via cyclooxygenase pathway, transforming Growth Factor Beta (TGF-β) singaling pathway and nitric oxide (NO) via NO synthases, and thereby inhibit the proliferation and activation of T cell and antigen presenting cells [14–17]. Since MSCs can be obtained from many sources in the body, a study compared canine bone marrow-derived stem cells (cBM-MSCs) and adipose-derived stem cells (cAD-MSCs) for their immunomodulatory efficacy. Interestingly, the two populations of MSCs showed comparable potential to suppress T cells, although TGF-β and adenosine signaling pathways seemed more important for cAD-MSCs while cyclooxygenase, TGF-β and adenosine signaling pathways all contribute to T-cell suppressive effect of cBM-MSCs [18].

Considering that immune-mediated KCS constitute a predominant part of clinical cases, the use of MSCs for KCS treatment has the potential to be an alternative therapeutic option. Accordingly, three independent groups performed intra-ocular, intra-lacrimal or intra-venous injection of allogeneic cAD-MSCs in KCS patients and showed significantly improvements in the tear production and other clinical signs [19–21]. Because repeated periocular transplantations of allogeneic MSCs in dogs were determined to be safe in vivo with systemic immunomodulatory effects [22], it is likely that topical application of cAD-MSCs could also exert similar therapeutic effect in KCS. In line with this speculation, topical application of MSCs successfully improved the tear production and tear film stability in an induced dry eye syndrome in rat model [23]. The aim of this study is to evaluate the therapeutic effect of topical application of cAD-MSCs in treating clinical canine KCS patients.

## Results

### Isolation and characterization of cAD-MSCs for iKCS treatment

**Fig. 1:**
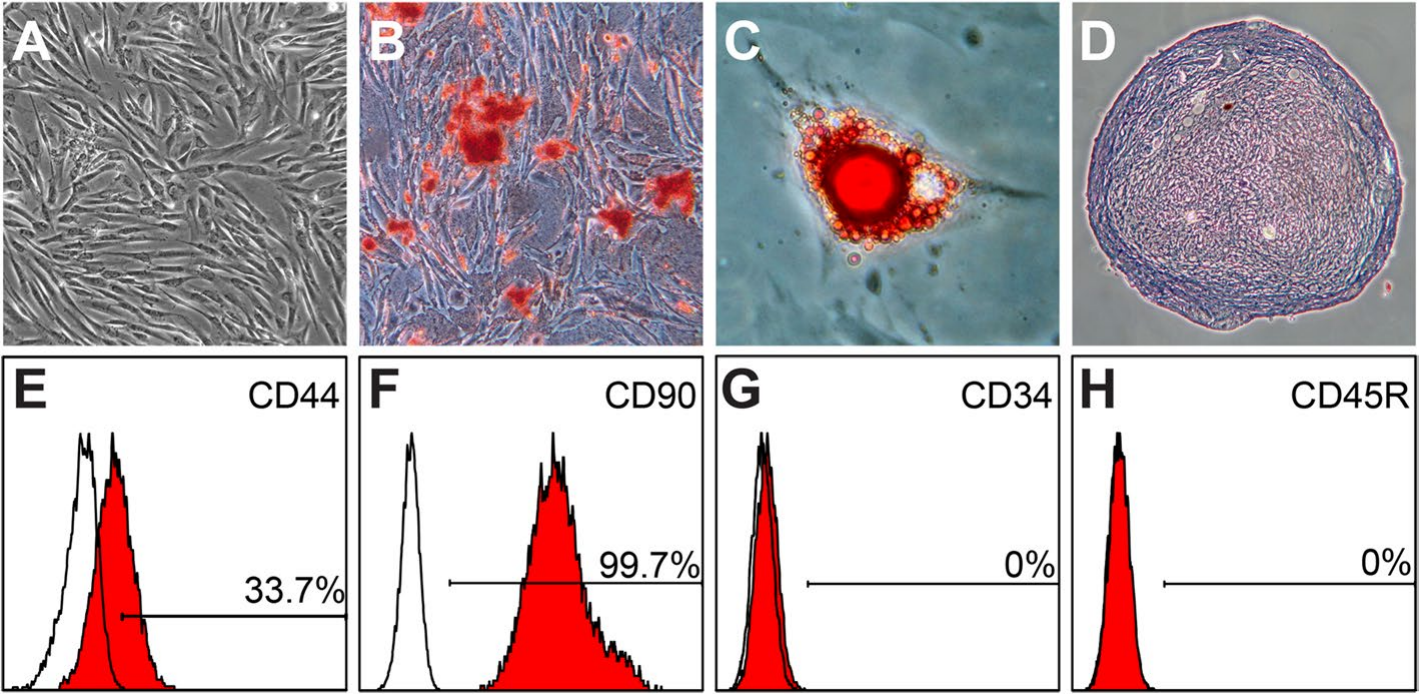
Characterization of cAD-MSCs used in this study. **A** The fibroblast-like cells with the ability to adhere to plastic culture plate were observed in isolated cAD-MSCs. **B** After osteogenic induction, Alizarin Red S staining demonstrated the mineralized extracellular matrix known as bone nodules. **C** After adipogenic induction, the lipid droplets stained positive with Oil Red O in differentiated cAD-MSCs. **D** After chondrogenic induction, the micromass was formed and sectioned. The tissue slides were stained with 0.1% of toluidine blue O and showed the typical glycosaminoglycans accumulation. **E**-**H** The flow cytometry showed that cAD-MSCs at passage 3 were moderately positive for CD44 (**E**) and predominantly positive for CD90 (**F**), while negative for CD34 (**G**) and CD45 (**H**)

Five clinically healthy female canine donors aged between 1.5 to 6 years old, weighted from 2 to 23 kg receiving ovariohysterectomy (OHE) were included for cAD-MSC donation. The cAD-MSCs were isolated and harvested successfully from omental adipose tissue from each donor. The isolated cells showed fibroblast-like morphology with the ability to adhere to plastic surface (Fig. 1A). Cells isolated from omental fat contained CFU and the number of CFU was 15.3 ± 2.3 (*n* = 6) at seeding density of 500 cells/cm^2^ and 12.0 ± 3.6 (*n* = 6) at seeding density of 300 cells/cm^2^, respectively. These cells formed white nodule-like colonies under osteogenic conditions for 7 days, and these white nodule-like colonies were strongly stained by ARS indicating successful osteogenic differentiation (Fig. 1B). Furthermore, the cells stopped expanding and began to fill with small vacuoles after 15 days in adipogenic induction medium. These small vacuoles were droplets of neutral lipids as stained by 0.5% ORO (Fig. 1C). In addition, the cells conformed into a spherical micromass on the second day in the chondrogenic induction medium. After 30 days cultured in chondrogenic induction medium, the micromass was sectioned and showed the typical glycosaminoglycans accumulation (pink-purple hue in the center) with 0.1% of toluidine blue O staining (Fig. 1D). The isolated cells were moderately expressed CD44 and predominantly negative for CD34 and CD45, while positive for CD90 at both passage 3 (Fig. 1E-H) and passage 7 (data not shown). Taken together, the cells isolated from omental adipose tissue during canine OHE were cAD-MSCs.

**Fig. 2:**
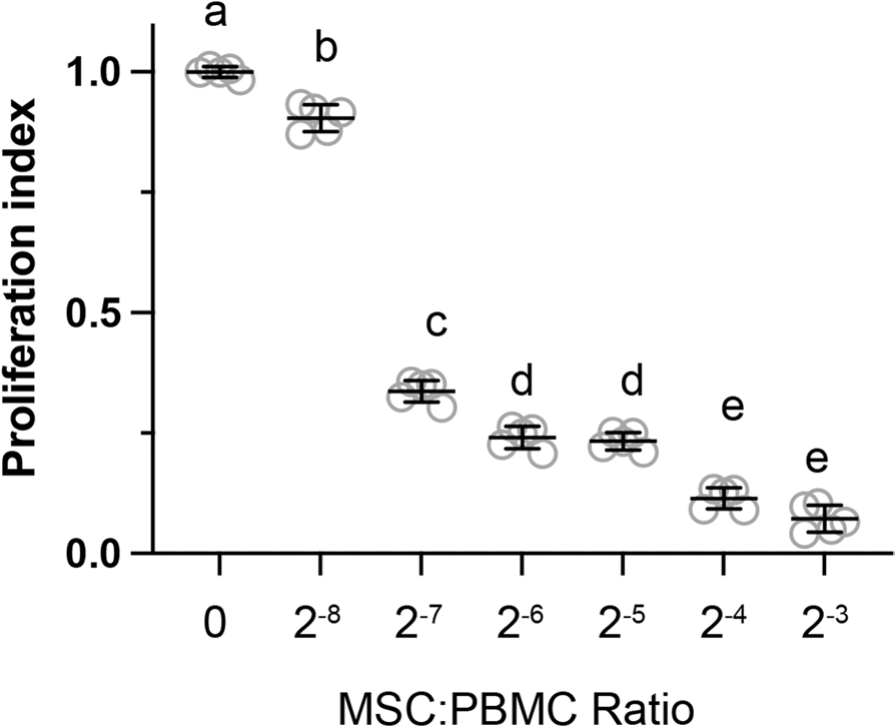
Immunosuppressive properties of cAD-MSCs. The PBMC showed significantly decreased proliferation index with the presence of cAD-MSC in a dose dependent manner. All data points were presented in clear circle and the mean with standard deviation was plotted for each group. Each letter indicated a distinct statistical groups

As iKCS are generally considered immune-mediated, it is key to test whether cAD-MSCs were immune-modulatory. The cAD-MSCs at passage 5 were subjected to mitogen-induced proliferation assay of PBMC. The PBMC proliferation index showed significantly decreased in all cAD-MSC treated groups and in a dose dependent fashion (Fig. 2). This result indicates that cAD-MSCs using in this study exert immunomodulatory activity and inhibit mitogenic responses of PBMCs.

To evaluate the feasibility of the topical and non-invasive method to treat canine iKCS patients with cAD-MSCs, we first evaluated the cell viability of cAD-MSC after prepared as eye drops. The eye drops stored at 4 °C in the dark showed 95% viability after 2 hours and remained 82% viability after 6 hours. On the other hand, the cell viability in cAD-MSCs eye drops stored at room temperature in the dark dropped to 92% at first hour and 81% after 4 h. By 5 h, the cell viability of cAD-MSCs in room temperature group dropped to 79%. Accordingly, all the clinical study results in this report used cAD-MSCs eye drops that were stored at 4 °C in the dark and topically applied to patients within 2 hours after preparation.

### Study population in the clinical trial

In total of 23 client-owned patient dogs were included in this study and received topical cAD-MSCs treatment with complete follow-up examinations. Based on previous treatment history, these patients were divided into two groups for analysis. Naïve group included 23 eyes of 12 dogs without history of immunosuppressant therapy (*n* = 23). Noticeably, one of patient was unilateral KCS. Refractory group included 21 eyes of 11 dogs that were unresponsive to previous immunosuppressant therapy (*n* = 21). One of the eyes was excluded because the eye was already enucleated due to refractory glaucoma secondary to lens luxation. Therefore, the total number of eyes evaluated in this study was 44. The age ranged from 5 to 15 years old in naïve group with a mean age at 7.91 ± 3.33 years old. The age ranged from 1 to 14 years old in refractory group with a mean age at 8.83 ± 3.79 years. Eleven dogs were spayed females, two were sexually intact females, nine were castrated males, and one was sexually intact males. Most subjects were purebred representing nine breeds and only one crossbred. The Maltese (*n* = 5) were the largest group of patients enrolled, followed by Dachshund (*n* = 4), Yorkshire terrier (*n* = 4), Poodle (*n* = 3) and Charles Spaniel (*n* = 1), Beagle (*n* = 1), Miniature Schnauzer (*n* = 1), Pug (*n* = 1), and Chihuahua (*n* = 1). The 11 patients in refractory group remained unresponsive to sustained immunosuppressant therapy at a mean period of 2 years and 4 months (ranged from 4 months to 5 years). Optimmune (*n* = 1), 1 to 2% CsA compounded in corn oil (*n* = 6) or 0.03% tacrolimus (*n* = 5) were administered in these patients as prior immunosuppressant. Information of each patient was presented in Tables 1 and 2.

**Table 1:** Information of each patient in naïve group

Age	Gender	Breed	STT		TBUT	
			OD	OS	OD	OS
13	NM	Beagle	0	0	0	0
15	NM	Miniature schnauzer	0	0	0	0
6	NF	Miniature dachshund	3	9	6	10
6	NF	Miniature dachshund	12	–	3	–
7	IF	Miniature poodle	6	6	3	3
7	NF	Miniature poodle	14	7	1	3
10	NM	Maltese	3	11	3	5
7	NF	Maltese	0	3	2	2
5	NF	Maltese	6	0	2	2
5	NM	Yorkshire terrier	12	9	3	2
6	IM	Yorkshire terrier	9	8	5	6

*NM* neutered male, *NF* neutered female, *IM* intact male, *IF* intact female, *OD* oculus dextrus, *OS* oculus sinister

**Table 2:** Information of each patient in refractory group

Age	Gender	Breed	STT		TBUT		Previous treatment / period
			OD	OS	OD	OS	
1	IF	Yorkshire terrier	10	–	3	–	2% CsA / 1Y
6	NM	mixed	17	21	11	9	2% CsA / 5Y
14	NM	Miniature dachshund	8	3	3	3	1% CsA / 3Y
5	NF	Pug	0	11	0	3	1% CsA / 4 M
14	NM	Yorkshire terrier	0	0	0	3	2% CsA / 2Y; TA / 1Y
8	NF	Maltese	0	3	3	5	TA / 1Y
9	NF	Maltese	0	4	2	2	TA / 3Y
10	NF	Maltese	15	5	3	3	1% CsA / 2Y
8	NM	King Charles Spaniel	0	0	3	0	Optimmune / 1Y
13	NM	Chihuahua	0	18	0	3	1% CsA / 2Y; TA/2 M
9	NF	Miniature dachshund	0	0	3	2	1% CsA / 3Y
9	NF	Miniature poodle	10	5	1	2	TA / 4Y

*NM* neutered male, *NF* neutered female, *IM* intact male, *IF* intact female, *OD* oculus dextrus, *OS* oculus sinister, *CsA* Cyclosporine A, *TA* Tacrolimus, *Y* years, *M* months

### The efficacy of topical cAD-MSCs

To test the efficacy of topical application of cAD-MSC eye drops for iKCS, ophthalmic examination results were statistically analyzed. All patients received topical treatment consisted of 2 × 10^6^ cAD-MSCs weekly for 6 consecutive weeks and complete ophthalmic examinations at baseline, 3rd, 6th and 9th week.

**Fig. 3:**
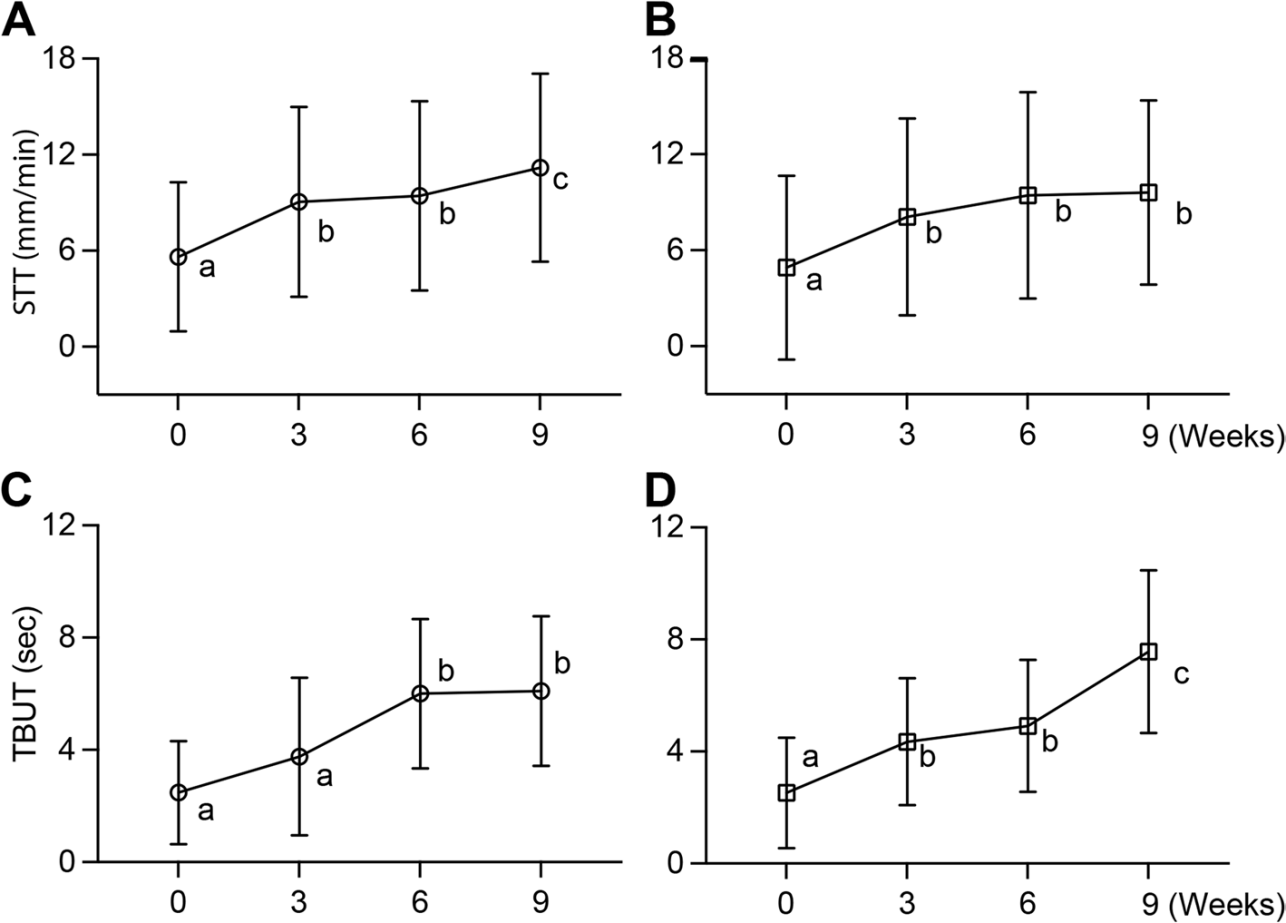
Effect of cAD-MSCs eye drops on clinical signs of KCS. **A**-**B** STT-1 were statistically increased in both naïve (**A**) and refractory (**B**) groups indicating the improvement in tear quantity. **C**-**D** TBUT were statistically increased in both naïve (**C**) and refractory (**D**) groups indicating the improvement in tear quality. The data were presented as mean with standard deviation, and each letter indicated a distinct statistical groups

The mean STT-1 values of each eye at baseline in the naïve group and refractory group were 5.62 ± 0.22 mm/min and 4.91 ± 0.25 mm/min. The mean STT-l value showed a significant increase at the third week (*P* < 0.01) and a further significant increase at ninth week (*P* < 0.05 compared to sixth week) (Fig. 3A). Interestingly, the mean STT-1 values increased in refractory group also showed a similar significant increase at the third week (*P* = 0.01) but the trend of improvement stalled thereafter (Fig. 3B). However, two-way ANOVA failed to detected significant variation between two groups (*P* = 0.612).

In this study, STT-1 values of each eye increased by more than 5 mm/min were defined as well responder. Accordingly, at the third week, 6 out of 21 eyes in naïve group and 7 out of 23 eyes in refractory group were well responder to cAD-MSCs. Nearly half of patients (10/21 and 11/23) in both groups showed well responses at sixth week. At the ninth week, the percentages of well-responders reached 57.1% (12/21) in naïve group and 56.5% (13/23) in refractory group (Table 3). However, similar to the STT-1 values, no statistically significant differences in response rates were detected between the two groups.

**Table 3:** Proportion of responders to treatment

Group	N	3rd week	6th week	9th week
Naïve	21	28.6%	47.6%	57.1%
Refractory	23	30.4%	47.8%	56.5%

Patients with STT-1 values increased more than 5 mm/min compared to pre-trial STT-1 values were defined as responders

Mean tear break-up time (TBUT) of each eye at baseline was 2.476 ± 0.087 seconds in naïve group patients, and significantly increased to 6 ± 0.12 seconds at sixth weeks, albeit the trend of improvement stalled thereafter (Fig. 3C). In the refractory group, the mean TBUT at baseline was 2.52 ± 0.086 seconds. Interestingly, this value significantly increased to 4.35 ± 0.10 seconds at the third week and showed a further significant increase to 7.57 ± 0.13 at ninth week (Fig. 3D). Similarly, two-way ANOVA failed to detect significant variation between two groups in TBUT (*P* = 0.663).

**Fig. 4:**
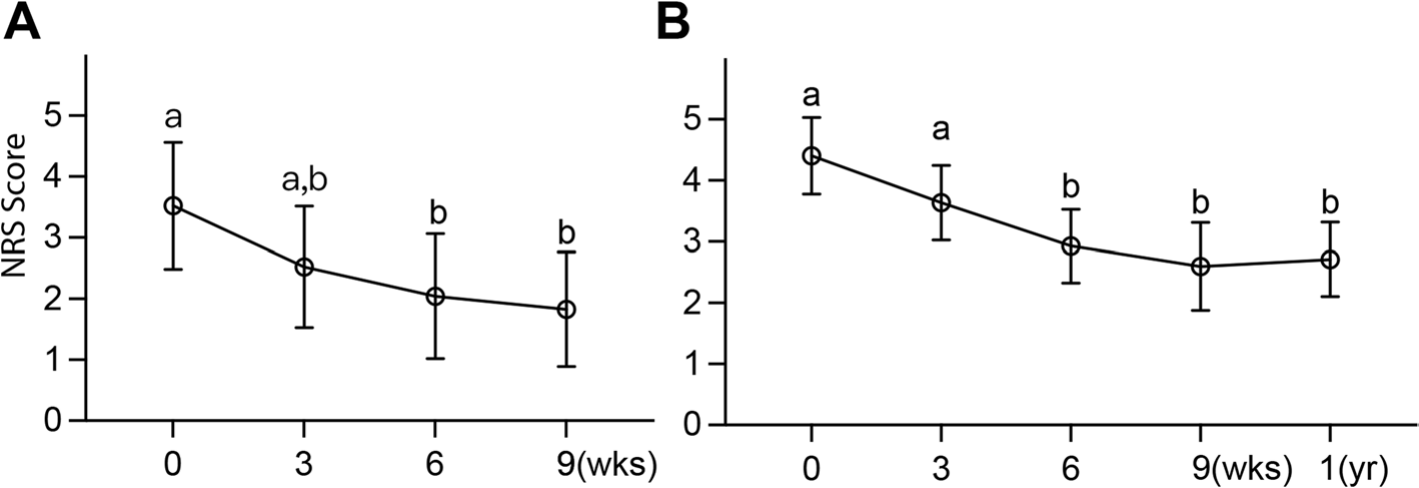
Effect of cAD-MSCs eye drops on clinical assessment. **A** The veterinarian assessment of clinical signs using NRS showed a statistically significant decrease after 6 weeks of administration of cAD-MSCs. **B** Owners assessment of clinical signs using NRS also showed a statistically significant decrease after 6 weeks of cAD-MSC treatment. The data represent the mean of hyperemia, discharge, corneal change and each letter indicated a distinct statistical groups

Clinical signs of all included patients were evaluated by the same clinician of the Ophthalmology Department and also observed by the owners. The numeric rating scale (NRS) score ranged from 1 to 5 was used to access severity of KCS signs. At baseline, the mean scores for signs of mucoid discharge, conjunctival hyperemia, and corneal change in all patients by the clinician and owners were 3.52 ± 0.045 (Fig. 4A) and 4.41 ± 0.027 (Fig. 4B), respectively. By the sixth week, the NRS score significantly decreased to 2.04 ± 0.043 (Fig. 4A) and 2.93 ± 0.027 (Fig. 4B) according to both clinician evaluation and owner observation. Interestingly, an one-year follow-up of clinical signs was obtained from each patient owner. The mean total scores for signs of mucoid discharge, conjunctival hyperemia, and corneal change in all patients observed by owners 1 year after the last cAD-MSC treatment was 2.71 ± 0.026. This score remained to be significantly lower than the baseline (*P* < 0.0001) and comparable to the score at ninth week (*P* > 0.9999) (Fig. 4B).

## Discussion

In this study, we demonstrated that non-invasive topical application of cAD-MSCs ameliorates canine iKCS. Although cAD-MSCs may exert comparable immunomodulatory effect as cBM-MSCs via slightly different mechanisms [18], there are several advantages to choose adipose tissue as the source of MSCs. One of the predominant reason to fail in obtaining cBM-MSC is bone marrow failure. Previous study showed that 30-40% of bone marrow aspiration from humerus and femur failed to harvest cBM-MSCs and most likely due to bone marrow failure in these bones [24]. On the contrary, cAD-MSCs were successfully harvested and isolated in all five omental adipose tissues collected in this study. Furthermore, the omental adipose tissues were collected during OHE procedures, which were unrelated to this study, and hence minimize the distress caused to the animal for the isolation of MSCs. In accordance with previous study that cAD-MSCs can be obtained from small amount of adipose tissue [25], 5 mL of adipose tissue yielded about 3 × 10^7^ cells in primary culture on average in this study. We further expand cAD-MSCs with a dilution factor of 1:2 to 1:3 in every passage. As we used of 2 × 10^6^ cells at passage 4 to 6 in every treatment, one harvest could easily provide enough cAD-MSCs for more than 120 treatments.

Although MSCs originally attracted attentions due to it multiple differentiation potentials, it is now generally acknowledged that transplanted MSCs do not persist in the host. Allogenic MSC-derived ectopic tissue engraftment hardly occur [26]. The “hit-and-run” model suggests that the various beneficial effects of MSC transplantation might be predominantly attributed to the paracrine effects, by which the MSCs interact with and alter the activities of host cells including the immune milieu [27]. In this study, the immunomodulatory activity of cAD-MSC was also demonstrated by mitogen-induced proliferation assay of PBMCs, and a dose-dependent response between MSC:PBMC ratios of 1:256 to 1:8 was observed (Fig. 2). Although fitted into different response models, similar results were also observed previously with a similar dose-dependent range in MSC:PBMC ratios [28, 29]. Interestingly, it is consistently found that the immunomodulatory effect of MSCs saturated when the MSC:PBMC ratio above 1:10. A healthy dog have about 4 × 10^7^ to 3.8 × 10^8^ lymphocytes per kilogram of the bodyweight. A 1:10 dose means at least 4 × 10^6^ MSCs per kilogram bodyweight for a systematic injection. As the canine KCS is now considered a regional autoimmune reactivity mediated by T cells and we introduced the MSCs by topical application, it is likely that the required dose might be lower to saturate the immunomodulatory effect. The ideal dose of MSCs might worth further investigations.

In this study, 2 × 10^6^ MSCs in PBS-based vehicle were administered topically as eye drops once per week for six consecutive weeks. The cAD-MSCs eye drops can be stored at 4 °C in the dark for more than 6 hours or at room temperature in the dark for more than 4 hours to maintain 80% viability. However, the costs and convenience to prepare cAD-MSCs eye drops remains to be a challenge. Techniques enabling the cAD-MSCs to be aseptically processed, frozen for shipping, storage, easily and quickly thawing at the point of clinical use were thoroughly discussed [30]. Ideally, the cAD-MSCs should be packaged into single-use vials or ampule. For instance, Cupistem (Anterogen, South Korea) is an adipose stem cell product that is used for the treatment of Crohn’s fistula, and the stem cells were suspended in DMEM and packaged into single-use vials containing 3 × 10^7^ cells/mL [31].

Several publications have appeared in recent years documenting that topical administration of MSCs could be effective for treatment of immune-mediated disease such as KCS. The safety of allogeneic canine adipose-derived mesenchymal stem cells was previously described. MSCs were delivered topically to healthy laboratory beagles, and the sequential examinations showed that repeated peri-ocular and intra-articular transplantations of allogeneic MSCs were safe [20, 22, 32]. However, periocular injection procedure requires sedation or general anesthesia, and may lead to iatrogenic or incidental injury during the injection process. In this study, we aimed to find a non-invasive, convenient, and efficacious route to treat canine KCS. To the best of our knowledge, there were no previous report evaluating the efficacy of topical allogenic cAD-MSCs in clinical canine KCS patients when this study was carried out (2018).

Following the introduction of CsA in 1987 [4], tacrolimus in 2005 [7] and pimecrolimus in 2009 [9], the refractory KCS declined dramatically. In 1990s, canine patients with a STT ≥ 2 mm/min have an approximately 80% chance of responding to 2% CsA with increased tear secretion, while those dogs with STT < 2 mm/min respond in approximately 50% of cases [33]. One clinical trial evaluated the efficacy of pimecrolimus eye drops by comparing with CsA in canine KCS patients. The result showed that 63 and 48% of dogs responded to pimecrolimus and CsA, respectively. Pimecrolimus (1%) oily eye drops are more effective than CsA ointment in controlling KCS in dogs [9]. However, the use of immunosupprants in KCS patients might be lifelong, and a recent study showed that the STT value in half of the KCS affected eyes dropped below 10 mm/min only about 21 days after treatment withdrawn [34]. Furthermore, a significant number of canine KCS patients do not respond to tear stimulant treatment. For those canine KCS patients which have proven to be refractory to all tear stimulant remain candidates for parotid duct transposition (PDT) surgery [35]. Interestingly, 56.5% of the patients who were unresponsive to prior immunosuppressant therapy showed improved tear volume following cAD-MSCs treatment in this study. Moreover, the improvement on clinical signs observed by owners maintained in one-year follow-up. Considering the convenience and financial implications, weekly administration of cAD-MSCs for 6 consecutive weeks can be a therapeutic option to increase the quantity and quality of tears and improved clinical signs in clinical canine KCS patients.

In our study, both refractory and naïve KCS patients responded to the treatement of cAD-MSCs in similar ways. This result implied that cAD-MSCs ameliorate the canine iKCS via distinct mechanisms from immunosuppressant eye drops. The immune suppressive pathways in canine MSCs have not been thoroughly studied, but a recent study showed that topical application of MSCs ameliorates inflammatory levels in KCS patient dogs indicated by reduced CD4, IL1, IL6 and TNFα levels [36]. It is generally agreed that canine iKCS is an autoimmune condition predominantly mediated by T-cell population [3]. Immune suppressant eye drops such as CsA, tacrolimus and pimecrolimus modulate T-cell response by blocking signaling pathways of T-cell receptor via calcineurine [37]. It is likely that MSCs involve in the modulation of T-cell response via multiple secretory pathways. For example, evidence showed that TGF-β signaling pathways and adenosine signaling are both involved in the suppression of T-cell activation by AD-MSCs [18]. Although cell-free therapeutic reagents are more appealing than MSCs. Application of simple MSC secreted materials might not work as effective as MSCs, since the immune modulatory activity of MSCs are dependent on the stimulatory immune milieu [38]. It is of interest to further investigate the varieties of immunomodulatory mechanisms of MSCs and the etiology of iKCS for the development of cell-free reagents. In addition, according to the result of this study, the combined administration of cAD-MSC and immunosuppressant eye drops might result in an even greater improvement.

## Conclusion

In conclusion, weekly administration of cAD-MSCs for 6 consecutive weeks increased the quantity and quality of tears and improved clinical signs in clinical canine KCS patients. Most patients appeared relieved from those irritation and uncomfortable signs, and thus their quality of life seemed improved following treatment. Our findings confirm the interest to develop cAD-MSCs as a potential therapy for canine KCS, especially for those unresponsive to immunosuppressant therapy.

## Methods

### Isolation of canine adipose-derived mesenchymal stem cells

All experimental procedures in this study were reviewed and approved by National Taiwan University Veterinary Hospital (Clinical Research No: 000030 and 000031) and the Institutional Animal Care and Use Committee of National Taiwan University (IACUC No: NTU106-EL-00097).

To obtain cAD-MSCs, fat donors were recruited from canine patients in National Taiwan University Veterinary Hospital (NTUVH) with the owners fully consented. The inclusion criteria of donors were modified from the canine blood donors as following [24, 39]: (1) between one to eight years old; (2) weight more than 5 kg and body condition scoring (BCS) 3/5 or above; (3) be up-to-date on vaccinations; (4) have no prior history of blood-borne illness; (5) be fit, healthy and not on medication. In addition, the recruited patients were already scheduled for laparotomy unrelated to this study and the adipose tissues were then harvested from omental adipose tissue using standard sterile surgical procedures during the laparotomies under general anesthesia [40].

The adipose tissue samples were collected into 10 mL pure medium [Dulbecco’s modified Eagle’s medium (DMEM; Gibco, Waltham, MA, USA) supplemented with 3.7 mg/mL sodium bicarbonate (Sigma-Aldrich, St. Louis, MO, USA), 100 U/mL penicillin and 100 g/mL streptomycin (Gibco)]. To isolate the cAD-MSCs [25, 41], each adipose tissue sample was weighed, trimmed, added with equal volumes of pure medium, and then digested with 1 mg/mL collagenase type IA (Sigma-Aldrich) for 1 hour at 37.5 °C. After the collagenase activity was neutralized, the sample was then centrifuged at 2000 RPM for 5 minutes to remove the mature adipocytes in the supernatant. The pellet was re-suspended in 10 mL of lysis buffer (0.15 M NH_4_Cl, 1 mM KHCO_3_, 0.1 mM Na_2_-EDTA, pH 7.3) and incubated for 10 minutes to remove red blood cells. The sample was then filtered through a 70-μm nylon cell strainer (Falcon, Durham, NC, USA), centrifuged at 2000 RPM for 5 minutes, and the pellet was re-suspended in 3 mL of culture medium [10% heat-inactivated fetal bovine serum (FBS; Hyclone, Logan, UT, USA) in pure medium]. The viable cells were estimated using a hemocytometer with 0.2% trypan blue staining (Gibco). The cells were seeded at a density of 1 × 10^4^ cells per cm^2^ with the culture medium replaced every 3 days until 70-80% confluence.

### Colony forming unit (CFU) assay

To analyze the colony forming efficiency of cAD-MSCs, the cells were incubated at two different seeding densities (350 and 500 cells/cm^2^) in culture medium for 10 days [42]. The cells were washed with PBS, fixed by methanol (Sigma-Aldrich) for 5 minutes, and then stained with KaryoMAX Giemsa (Gibco) for 20-30 minutes. The cells were rinsed in deionized water and colonies with a 2-mm or larger diameter were counted as a colony-forming unit (CFU).

### Tri-lineage differentiation of cAD-MSCs

To confirm the multipotent differentiation potentials, the cAD-MSCs were analyzed for their capacity to differentiate into adipogenic, chondrogenic, and osteogenic lineages [24, 25, 41, 43]. For osteogenic differentiation*,* the cells were plated at the seeding density of 1000 cells/cm^2^ and cultured in osteogenic induction medium (culture medium supplemented with 50 μM L-ascorbic acid 2-phosphate, 10 mM β-Glycerophosphate and 0.1 μM dexamethasone) for approximately 7-10 days with the osteogenic induction medium changed every 3 days. Osteogenesis was demonstrated by extracellular mineralized calcium phosphate deposits, which was assessed by staining the cells with 40 mM Alizarin Red S (ARS; Sigma-Aldrich) for 15 minutes followed by washing with PBS and fixing in 10% formaldehyde (Sigma-Aldrich).

For chondrogenic differentiation, each set of 2.5 × 10^5^ cells was centrifuged at 300×*g* for 5 minutes, and the cells were cultures in chondrogenic induction medium [DMEM supplemented with 1% FBS, 10 ng/mL of transforming growth factor beta 1 (TGF-β1; R&D Systems, Minneapolis, MN, USA), 6.25 μg/mL of insulin (Sigma-Aldrich) and 50 nM of L-ascorbic acid − 2 phosphate] for up to 30 days with the chondrogenic induction medium changed every 3 days. To assess the chondrogenic differentiation, the spherical micromasses were fixed in 10% formaldehyde for 1 hour and prepared into paraffin sections in 6-μm thickness. After dewaxing and rehydration, the slides were stained with 0.1% of toluidine blue O (Sigma-Aldrich) for 5 minutes, washed with PBS, mounted in mounting medium (Leica Biosystems, Wetzlar, DE) and documented for the sulfated glycosaminoglycans (GAG) accumulation under a microscope (DM2500, Leica Microsystems, Wetzlar, DE).

For adipogenic differentiation, the cells were plated at the seeding density of 10,000 cells/cm^2^ to for amplification until confluence and then were transferred to adipogenic induction medium (culture medium supplemented with 1 μM dexamethasone, 10 μg/mL insulin, 100 μM indomethacin and 0.5 μM isobutyl-methylxanthine) for 35-40 days with the adipogenic induction medium changed once every 3 days. The cells were fixed in 10% formaldehyde for 10 minutes, dehydrated with 1 mL of propylene glycol (JT Baker, Phillipsburg, NJ, USA) for 1 minute, and then immersed in 200 μL of 0.5% Oil Red O (ORO) (Sigma-Aldrich) for 15 minutes to visualize the lipid droplets. After the staining was stopped with 1 mL of 60% propylene glycol, the cells were washed with deionized water and assessed under a microscope.

### Immunophenotyping of cAD-MSCs

To evaluate the immunophenotypes of isolated cAD-MSCs [24], the cells were mixing with antibody and stored at 4 °C in the dark for at least 30 minutes. After washing with PBS with 2% FBS, the cells were fixed in PBS with 1% FBS and 3% formaldehyde, the cells were then analyzed for the expressions of surface markers by flow cytometry (FC500, Beckman-Coulter, Fullerton, CA). The fluorescence-conjugated or isotype control antibodies used in this study were as listed (Table 4).

**Table 4:** The antibodies used in flow cytometric analysis

Antibody	Clone	Vendor	Dose (/test)	Isotype
CD44-FITC	YKIX337.8	eBioscience, San Diego, CA, USA	0.025 μg	IgG2a
CD90-PE	YKIX337.217		0.1 μg	IgG2b
CD34-PE	1H6		0.05 μg	IgG1
CD45R-PE	YKIX753.22.2		0.0015 μg	IgG2b
IgG2a-FITC (Rat)	eBR2a		0.1 μg	
IgG1-PE (Mouse)	P3.6.2.8.1		0.025 μg	
IgG2b-PE (Rat)	eB149/10H5		0.1 μg	

### Immunomodulatory effect of cAD-MSCs

To estimate the immunomodulatory activities of cAD-MSCs, the mitogen-induced proliferation assay of peripheral blood mononuclear cells (PBMCs) was adopted from previously published methods [44, 45]. Breifly, canine whole blood was collected in ethylenediaminetetraacetic acid (EDTA) tubes (BD, Franklin Lake, NJ, USA) and centrifuged at 1200 RPM for 10 minutes. The cells were re-suspended with pre-warmed RPMI-1640 medium (Gibco) containing 20% FBS, 100 U/mL penicillin, 100 μg/mL streptomycin, 50 μM 2-mercaptoethanol (Sigma-Aldrich) and 55 μM Ficoll-Paque (GE Healthcare, Chicago, IL, USA). The mononuclear cells were separated by cell density gradient centrifugation. The centrifugation was started at 500 RPM and increased 100 RPM every 30 seconds till 1700 RPM for 20 minutes.

After washed with RPMI-1640 medium, 1 × 10^5^ of the resulting peripheral blood mononuclear cells (PBMCs) were seeded in triplicate with the addition of a 2-fold serial dilution of the mitomycin C (10 μg/mL; Sigma-Aldrich) treated cAD-MSCs (in the dark at 37 °C for 2 hours) in 96-well plates (TPP, Trasadingen, Switzerland) followed by mitogenic stimulation using 5 μg/mL concanavalin-A (ConA; Sigma-Aldrich). The cAD-MSC only samples as well as PBMCs with or without ConA stimulation were also prepared to calculate the proliferation indexes. After 4 days in culture, the total viable cell number was analyzed by using Cell Counting Assay Kit-8 (CCK-8; Dojindo Molecular Technologies, Rockville, MD, USA) according to the manufacturer’s protocol. The absorbance at 450 nm was read by an ELISA plate reader (ThermoMax, Molecular Devices, San Jose, CA, USA). The proliferation indexes of PBMC with conA relative to without conA was considered 100% and all sample were normalized in the same fashion after the signal from cAD-MSC only control were subtracted.

### Preparation of cAD-MSCs eye drops

To prepare cAD-MSCs into eye drops for dogs [23, 46], the cAD-MSCs at passage 4 to 6 were revived 2 hours before topical application, re-suspended at the concentration of 2 × 10^6^ cells/50 μL PBS solution, and then aseptically filled into sterilized 1-mL disposable syringes (Terumo, Tokyo, JP). To assess the qualities of the cAD-MSCs in the eye drops under various storage conditions, 10 μL of eye drops were gently mixed with equal volume of 0.4% trypan blue and the percentages of viable cells were counted on a hemocytometer. For topical application, the AD-MSCs from different donors were not pooled but were also not segregated for each recipient.

### Inclusion and exclusion criteria of patients for the clinical trial

All subjects in this study were clinical canine patients of NTUVH admitted between March 2018 to September 2018 regardless of gender, breed or age. All patients received a complete ophthalmic examination to confirm the diagnosis of KCS by the same veterinarian (LNW) of the Ophthalmology Department. All patient-owners were fully informed the pathogenesis, clinical signs and traditional treatments of KCS as well as the safety and the course of topical cAD-MSCs treatment before entering this study. Diagnosis of KCS in the canine patients were based on the medical history, clinical signs and standard measurements of tear production using the Schirmer tear test-1 (STT-1) (≤ 15 mm/min) [47]. The subjects with other ocular diseases that could affect the result of this study such as corneal ulceration, or with other known causes for KCS were excluded from this study (Table 5). In addition, subjects topically or systemically treated by any of the following drugs within 14 days before the study were also excluded from this study: corticosteroids, atropine, pilocarpine, sulfa-containing drugs and immunosuppressants such as CsA, pimecrolimus, or tacrolimus. As the majority of the subjects used artificial tear routinely before joining this study, the subjects kept using the same type and frequency of artificial tear after they joined this study to ensure that AD-MSC treatment was the only variation factor of the KCS patients between before and after joining the clinical trial.

**Table 5:** Exclusion of patients with other known causes for KCS

Causes of KCS	Considerations in the diagnostic process
Congenital	onset of clinical signs less than 6-month-old STT ≤ 5 mm/min absence of other known causes of KCS lack of response to topical immunosuppressant
Metabolic	Blood exam/clinical signs
Infectious	History/PCR/blood exam/biopsy
Drug induced	History
Neurogenic	Physical exam (ipsilateral dry nose) Neurological exam(±facial paralysis, Horner’s syndrome)
Iatrogenic	History

This is a prospective study aiming to evaluate the therapeutic effect of topical cAD-MSCs application in canine KCS. In considering the welfare of the clinical patients, negative control group was omitted in this study as canine KCS progresses robustly without treatment [4, 7]. The therapeutic effect was estimated by comparing the treated results to the baseline conditions. As AD-MSCs can inhibit T-cell activation and proliferation [14–17], it is intriguing whether cAD-MSCs trigger the same therapeutic pathway as commonly used immunosuppressants and therefore might show less significant therapeutic effect to refractory KCS patients. To further investigate the effect of AD-MSCs on refractory KCS patients, the subjects were segregated into two groups: naïve group included subjects without any previous immunosuppressant treatment, and refractory group included subjects unresponsive to prior immunosuppressant treatment. In accordance with the exclusion criteria, all subjects in refractory group had stopped immunosuppressant treatments for at least 14 days.

### Trial protocol and ophthalmic examinations

**Fig. 5:**
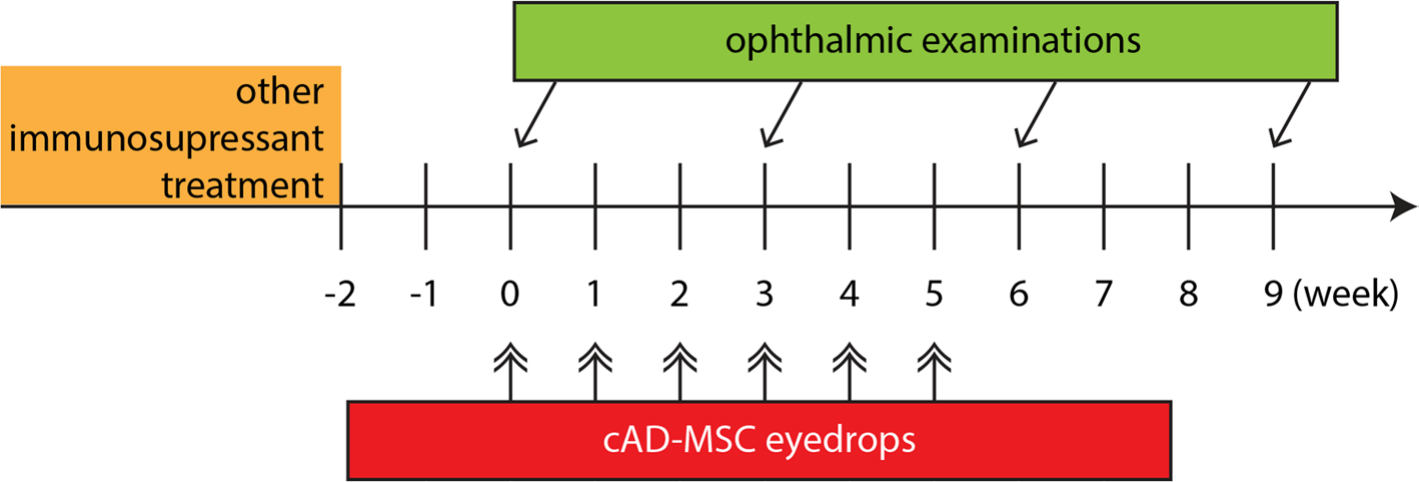
Schematic diagram of the clinical trial. All patients received weekly cAD-MSCs eye drops with 2 × 10^6^ cells suspended in 50 μL PBS for 6 consecutive weeks. Participants took four complete ophthalmic examinations at baseline (0), 3rd, 6th and 9th week. The patients in refractory group were confirmed that had stopped applying immunosuppressant for at least 14 days

All subjects received a drop of 50 μL PBS containing 2 × 10^6^ cAD-MSCs during weekly outpatient appointment for 6 consecutive weeks (Fig. 5). A complete set of ophthalmic examinations was performed before (baseline), during (third and sixth week) and after treatment (ninth week) respectively (Fig. 5). To quantify the clinical signs of the patients, a five-point NRS was designed to describe the severity of mucoid discharge, conjunctival hyperemia, and corneal changes by both subject owners and the veterinarian. For veterinarian, the NRS was determined by using a slit-lamp biomicroscopy. The standard for NRS scores ranged from 1 to 5 were as listed (Table 6) [48]. All subject owners were also required to submit a NRS scoring result at ninth week before the disclosure of prior ophthalmic examination results. The NRS from subject owners were queried again from medical records or by telephone at least 1 year after the end of the topical cAD-MSCs therapy.

**Table 6:** The numeric rating scale (NRS) of clinical signs

Mucoid discharge	5 = severe	Mucopurulent crust, stinky
	4 = advanced	Yellowish, mucoid discharge, smelly
	3 = moderate	Yellowish, sticky discharge
	2 = lightly	Clear, but discharge volume increased
	1 = normal	Absent discharge
Conjunctival hyperemia	5 = severe	Diffuse beefy red
	4 = advanced	Individual vessels not easily discernible
	3 = moderate	More diffuse, deeper crimson red
	2 = lightly	Vessels definitely injected above normal
	1 = normal	Vessels normal
Corneal change	5 = severe	Opaque, iris invisible with pigmentation, keratitis
	4 = advanced	Opalescent areas, no details of iris visible
	3 = moderate	Easily discernible translucent area, details of iris slightly obscured
	2 = lightly	Scattered or diffuse areas, details of iris clearly visible
	1 = normal	No opacity

The NRS score was a five-point scale used to access the severity of mucoid discharge, conjunctival hyperemia, and corneal changes by dog owners and the veterinarian

A complete set of ophthalmic examinations consists of three tests. (1) Schirmer tear test-1 (STT-1) was performed with Color Bar Schirmer tear strip (Eagle Vision, Memphis, TN, USA) to assess the aqueous tear production [49]. STT-1 value equal or less than 15 mm/min was considered abnormal. (2) Tear break-up time (TBUT) was performed using fluorescein paper (Haag-Streit AG, Köniz, Switzerland) and a slit-lamp biomicroscopy (Sl-17, KOWA, Tokyo, Japan) to assess the stability of tear film. TBUT less than 15 seconds was considered abnormal [50]. (3) A NRS scoring system was also implemented in this study to quantify the severity of clinical symptoms.

### Statistical analysis

The proliferation indexes resulted from the mitogen-induced proliferation assay of PBMCs were evaluated by one-way ANOVA with Bonferroni test. The intragroup comparisons of STT-1 and TBUT were analyzed by repeated-measures analysis of variance (ANOVA) and followed by Bonferroni test. The comparisons of STT-1 and TBUT between groups were carried out by using two-way ANOVA, and the response rates between two groups were evaluated by Mann-Whitney test. The assessment of clinical signs using NRS by owners and veterinarian was analyzed using Friedman test, a non-parametric one-way ANOAVA for repeated measurements, and followed by Dunn’s test for multiple comparisons. A *P*-value less than 0.05 was considered statistically significant. All data were presented as mean ± standard deviation (SD).

## Data Availability

All data and necessary information are included in this report.
